# Subunit composition of α5-containing nicotinic receptors in the rodent habenula

**DOI:** 10.1111/j.1471-4159.2012.07714.x

**Published:** 2012-05

**Authors:** Petra Scholze, Gabriele Koth, Avi Orr-Urtreger, Sigismund Huck

**Affiliations:** * Center for Brain Research, Medical University of ViennaVienna, Austria; † Tel-Aviv Sourasky Medical Center and Sackler School of Medicine, Tel Aviv UniversityTel Aviv, Israel

**Keywords:** gene deletion, habenula, interpeduncular nucleus, nicotine abuse, nicotinic acetylcholine receptor, subunit composition

## Abstract

Gene association studies in humans have linked the α5 subunit gene *CHRNA5* to an increased risk for nicotine dependence. In the CNS, nicotinic acetylcholine receptors (nAChRs) that contain the α5 subunit are expressed at relatively high levels in the habenulo-interpeduncular system. Recent experimental evidence furthermore suggests that α5-containing receptors in the habenula play a key role in controlling the intake of nicotine in rodents. We have now analysed the subunit composition of hetero-oligomeric nAChRs in the habenula of postnatal day 18 (P18) C57Bl/6J control mice and of mice with deletions of the α5, the β2, or the β4 subunit genes. Receptors consisting of α3β4*^1^ clearly outnumbered α4β2*-containing receptors not only in P18 but also in adult mice. We found low levels of α5-containing receptors in both mice (6%) and rats (2.5% of overall nAChRs). Observations in β2 and β4 null mice indicate that although α5 requires the presence of the β4 subunit for assembling (but not of β2), α5 in wild-type mice assembles into receptors that also contain the subunits α3, β2, and β4.

Neuronal nicotinic acetylcholine receptors (nAChRs) are pentameric ion channels consisting of five identical (homopentameric) or different (heteropentameric) subunits. The predominant hetero-oligomeric nAChRs in the CNS contain the subunits α4β2, whereas α3β4 prevail in the PNS (McGehee and Role 1995). However, the presence of the additional subunits α2, α5, α6, and β3 in distinct regions of the nervous system gives rise to a much larger variety of receptors (Gotti *et al.* 2006). Although the reason for such diversity is unknown it provides the possibility to develop therapeutic nicotinic ligands that target specific types of receptors.

Unlike most of the other α subunits, α5 stands out as it always requires the presence of at least one other α, along with β2 or β4 (Ramirez-Latorre *et al.* 1996; Fucile *et al.* 1997; Gerzanich *et al.* 1998). We have recently reported that in the superior cervical ganglion (SCG) of wild-type (WT) C57Bl/6J mice, α5 assembles only into α3β4 receptors (David *et al.* 2010). Consequently, all α5-containing receptors are lost in the SCG of mice lacking the β4 subunit. These results are in keeping with the rat SCG, where about 25-30% of heteromeric nAChRs are of the α3β4α5 type (Mao *et al.* 2006). However, several parts of the CNS such as the hippocampus, the striatum, the cerebral cortex, or the thalamus, express receptors that contain α5 in combination with the subunits α4 and β2 (Mao *et al.* 2008). Yet, in the rodent habenulo-interpeduncular system (the habenular complex), α5 reportedly co-assembles not only with β2 but also with β4 (Grady *et al.* 2009).

Earlier efforts to identify gene polymorphisms that may lead to nicotine addiction have highlighted the *CHRNA5*/*CHRNA3* gene cluster on chromosome 15 as a potential candidate (Berrettini *et al.* 2008; Bierut *et al.* 2008). Hence, allelic variations in the α5 subunit gene which result in a decreased function of receptors increase vulnerability to tobacco addiction (Bierut *et al.* 2008). The brain region primarily accountable for this may be the habenulo-interpeduncular system. Its anatomical connections allow the habenula to act as a node to link the forebrain to the midbrain regions that are involved in regulating emotional behaviors such as pain, stress, and anxiety (Hikosaka 2010). In fact, recent observations assign α5-containing nAChRs in the habenular complex a key role in controlling nicotine consumption (Fowler *et al.* 2011; Frahm *et al.* 2011) and nicotine withdrawal (Salas *et al.* 2009). α5 may require the presence of the β4 subunit, because decreased signs of nicotine withdrawal have been observed not only in mice lacking α5 but also in β4 knockout (KO) animals (Salas *et al.* 2004, 2009; De Biasi and Salas 2008).

As the pharmacological and biophysical properties of α4β2* differ significantly from α3β4* receptors (McGehee and Role 1995), knowledge of subunits that co-assemble with α5 is important for understanding the role of habenular nAChRs in nicotine abuse and dependence. We therefore re-analysed nAChRs found in the habenula of rats and mice and paid particular attention to α5-containing receptors.

## Materials and methods

### Generation and purification of antibodies

All antibodies were targeted against the cytoplasmic loop region of mouse nAChR subunits as previously published for anti-α3, anti-α4, anti-α5, anti-β2, and anti-β4 (David *et al.* 2010). The immunoprecipitation (IP) efficacy and specificity of our anti-α3, -α4, -β2, and -β4 antibodies has previously been tested with recombinant receptors expressed in HEK-293 cells, and by comparing the IP results in the SCG of α5β2 and α5β4 double KO (Kedmi *et al.* 2004) mice (which express pure α3β4 and α3β2 receptors, respectively) with polyethyleneglycol precipitation of all solubilized receptors. Furthermore, we took advantage of nAChR-KO mice to exclude false-positive reactions of our anti-α5, -β2, and -β4 antibodies (Figure S1, David *et al.* 2010). We now probed the efficacy of the anti-α5 antibody, which was generated by immunizing rabbits with the loop region of the α5-subunit, on receptors generated by replacing the cytoplasmic loop of the β2 subunit amino acids (aa) 345–415 by the loop of α5 (aa 362–418), and by co-expression of this chimera with α4 in HEK-293 cells (see Figure S1).

The proteins used for immunizing rabbits against the subunits α2 and α6 consisted of maltose-binding protein, fused to loop regions covering aa 361–444 (anti-α2) and aa 359–432 (anti-α6), respectively. The antibodies were affinity-purified using the corresponding glutathione S-transferase fusion protein coupled to Affi-Gel 10 (Bio-Rad Laboratories, Hercules, CA, USA) and probed with recombinant receptors expressed in HEK-293 cells as well as with native materials taken from the cerebellum, the *C. striatum*, and the interpeduncular nucleus (Figure S1).

### Animals and preparation of habenula

Experiments were performed on WT C57Bl/6J mice, and on mice with deletions of the nAChR subunit genes α5 (Wang *et al.* 2002), β2 (Picciotto *et al.* 1995), and β4 (Kedmi *et al.* 2004). β2 KO mice were generously provided by J.-P. Changeux (Pasteur Institute, Paris), α5 KO and β4 KO by Avi Orr-Urtreger (Sourasky Medical Center, Tel Aviv). Mice used in this study were backcrossed into C57Bl/6J background for 6 (β4), 7 (α5) or 12 (β2) generations after germ line transmission.

Sprague–Dawley rats (*Oncins France strain* A) were obtained from the Institute of Biomedical Research, Medical University of Vienna (Himberg, Austria) and bred in-house. All animals were kept in thermo stable rooms (21°C) on a light–dark schedule of 10 : 14 h in group cages with food and water freely accessible. Animal care and experiments are in accordance with the European Communities Council directive (86/609/EEC) and the Austrian federal law governing animal experimentation (Tierversuchsgesetz TVG 501/1989).

The great majority of experiments was performed with pre-pubertal mice and rats (mostly P18, range 17–19 days), killed by decapitation. Adult mice (6–8 weeks) and rats (300–600 g) were deeply anesthetized with diethylether before decapitation. We dissected entire habenulae, though hetero-pentameric nAChRs are highly enriched in the medial habenula (MHb) with only few such receptors in the lateral habenula (Clarke *et al.* 1985; Wada *et al.* 1989; Perry and Kellar 1995; Le Novere *et al.* 1996; Perry *et al.* 2002; Whiteaker *et al.* 2002). Habenulae were collected in Ca^2+^-free Tyrode’s solution: 150 mM NaCl, 4 mM KCl, 2.0 mM MgCl_2_, 10 mM glucose, and 10 mM HEPES, pH 7.4. After removal of the Tyrode’s solution, tissue was flash-frozen with liquid nitrogen and stored at -80°C for later use.

### Immunoprecipitation of [^3^H]-epibatidine labeled receptors

Receptors were solubilized in 2% Triton X-100 lysis buffer: 50 mM Tris–HCl pH = 7.5, 150 mM NaCl, 2% Triton X-100, supplemented with one complete mini protease inhibitor cocktail tablet (Roche Molecular Biochemicals, Indianapolis, IN, USA) per 10 mL buffer. Following one ultrasound pulse of 5 s duration at 30% energy level, samples were left for 2 hours at 4°C and thereafter centrifuged at 16 000 *g* for 15 min at 4°C. 130 μL clear supernatant from 0.5 habenulae (rat), 1.5 habenulae (WT, β2 KO), or 4 habenulae (β4 KO), respectively, were incubated with 20 μL 10 nM [^3^H]-epibatidine and 7 μg antibody in 30 μL phosphate-buffered saline (10 mM Na_2_HPO_4_, 1.8 mM KH_2_PO_4_, 2.7 mM KCl, 140 mM NaCl, pH = 7.4) on a shaking platform at 4°C over night. Non-specific binding was determined by adding 300 μM nicotine to half of the samples.

Heat-killed, formalin-fixed *Staphylococcus aureus* cells carrying protein A (Standardized Pansorbin-cells; Calbiochem, San Diego, CA, USA) were centrifuged at 2300 *g* for 5 min at 4°C. Pansorbin-pellets were washed twice with IP-High (50 mM Tris–HCl pH = 8.3, 600 mM NaCl, 1 mM EDTA, 0.5% Triton X-100), once in IP-Low (50 mM Tris–HCl pH = 8.0, 150 mM NaCl, 1 mM EDTA, 0.2% Triton X-100), and re-suspended with IP-Low. Twenty microliters of this suspension of Pansorbin cells were added to the above-mentioned cocktail containing the antibody, solubilized receptors, and [^3^H]-epibatidine for 2 h at 4°C on a shaking platform. Samples were centrifuged at 2300 *g* for 5 min at 4°C and washed twice with IP-High and once with IP-Low at 2300 *g* for 1 min at 4°C. Pellets were re-suspended in 200 μL 1 M NaOH and subjected to liquid scintillation counting. For sequential immunoprecipitation (IP) experiments, the supernatant was saved, and precipitated with a second antibody as described above.

### Quantification of protein contents in membrane preparations and lysates

All protein quantifications were performed using the BCA Protein Assay Reagent Kit (Pierce, Rockford, IL, USA) following the manufacturer’s instructions.

### Reagents

General chemical reagents were from Merck-VWR-Jencons, Radnor, PA. Substances not explicitly mentioned were from Sigma-Aldrich (St Louis, MO, USA).

### Data analysis

All data are presented as means ± SEM. Statistical analyses was performed with GraphPad Prism version 4.0 (GraphPad Software Inc., San Diego, CA, USA). Student’s *t*-test or one-way analysis of variance (anova), followed by Bonferroni’s multiple comparison test, were performed when appropriate.

## Results

### Antibodies for IP assays

Subunit-specific antibodies are essential pre-requisites for the analysis of the subunit composition of nAChR subtypes. The generation of antibodies directed against the subunits α3, α4, α5, β2 and β4 has been described in our previous publication (David *et al.* 2010). Anti-α2 and anti-α6 antibodies were newly generated. For a detailed characterization of antibodies, see the Methods section, Figure S1, and supplemental Materials provided in David *et al.* (2010).

### nAChRs in the mouse and rat habenula have similar subunit profiles

**Fig. 1:**
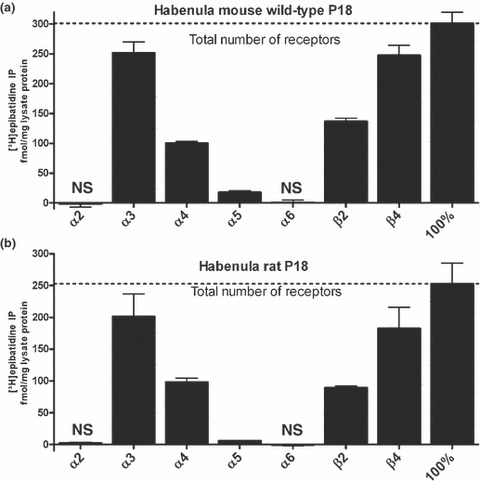
The overall number of nAChRs and the occurrence of distinct subunits are similar in the mouse and rat habenula. nAChRs from habenula of wild-type P18 mice (a) or P18 rats (b) were solubilized, labeled with 1 nM [^3^H]-epibatidine and immunoprecipitated with each of the subunit-specific antibodies indicated at the abscissa. Non-specific binding was measured in the presence of 300 μM nicotine and subtracted from the overall to obtain the specific binding shown in the figure. The mouse data represent means ± SEM of 3 (α2 and α6), 10 (α3), 7 (α4), 9 (α5), 23 (β2), or 21 (β4) independent experiments, each performed with duplicate or triplicate measurements. The rat data represent means ± SEM of three independent experiments, each performed with duplicate or triplicate measurements. The 100% values are determined by the combined use of anti-β2 plus anti-β4 antibodies, a protocol which precipitates all hetero-oligomeric receptors. NS: not significantly different from zero (*p* > 0.05, one sample Student’s *t*-test).

We assess the overall number of [^3^H]-epibatidine binding sites by the combined use of anti-β2 plus anti-β4 antibodies (David *et al.* 2010). Hence, IPs with a combination of the two antibodies will show 100% of heteropentameric receptors. We found the overall number of receptors was similar in P18 mice and rats (301.3 ± 18.4 fmol/mg lysate protein in mice, *n* = 13; 252.8 ± 32.7 fmol/mg lysate protein in rats, *n* = 3, Fig. 1). In addition, the subunit profiles of nAChRs in the habenula of WT mice and rats are very much alike (Fig. 1).

The majority of receptors of both species contain the subunits α3 (84% in mouse and 80% in rat) and/or β4 (82% of nAChRs in mouse and 72% in rat, Fig. 1). In either rats or mice, 33–45% of receptors contain the subunits α4 and/or β2 (Fig. 1). Given that more β2 than β4-containing receptors were found in the adult rodent habenula in a previous publication (Grady *et al.* 2009) we checked in mice whether the β2/β4 proportion might be developmentally regulated. In fact, when the numbers of β2- were compared with β4-containing receptors in one and the same assay, their rate of occurrence increased from 0.58 ± 0.03 (means ± SEM, *n* = 22 assays) in P18 mice to 0.88 ± 0.06 (means ± SEM, *n* = 6 assays) at 6–8 weeks old animals (significantly different *p* < 0.01, Student’s *t*-test). Hence, we find that β4-containing receptors outnumbered β2 not only in P18 but also in adult mice.

A small percentage of receptors include the accessory subunit α5 (6% in mouse and 2.5% in rat, both values significantly different from zero, *p* < 0.01 for rats and *p* < 0.001 for mice, one sample Student’s *t*-test; and both values significantly differ from each other, *p* < 0.05, one-way anova followed by Bonferroni’s *post hoc* multiple comparison test). Again, significantly higher levels of α5-containing receptors (about 27% of overall) have been reported particularly in the adult rat by Grady *et al.* (2009). When analysing the number of α5-containing receptors in adult rats we found a moderate increase from 2.50 ± 0.34% at P18 (means ± SEM, *n* = 3 assays) to 3.25 ± 0.11% (*n* = 7 assays) of overall receptors in adult rats (significantly different *p* < 0.05, Student’s *t*-test). We did not detect measurable amounts of the subunits α2 and α6 in either species (levels not significantly different from zero, *p* > 0.05, *n* = 3, one sample Student’s *t*-test; Fig. 1).

### Most receptors in the mouse habenula consist of α3β4*

**Fig. 2:**
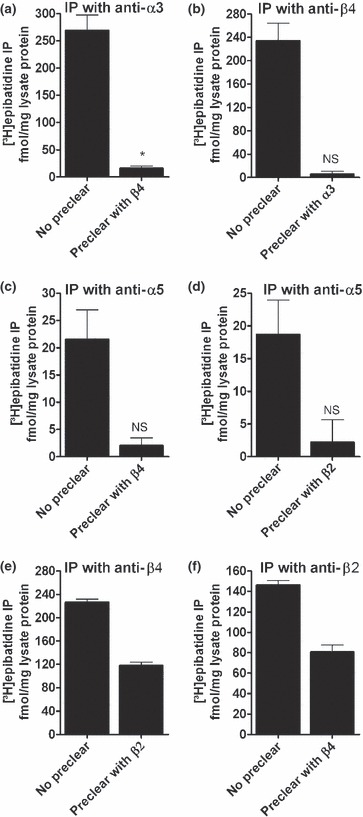
α3 only co-assembles with β4, and all α5 subunits assemble into α3α5β4β2-receptors in the mouse habenula. Wild-type mouse habenula extracts were first immunoprecipitated with the ‘clearing’ antibodies indicated at the abscissa (the ‘clearing’ antibody, see Mao *et al.* 2008). The resulting supernatants were then immunoprecipitated with antibodies against α3 (a), α5 (c, d), β2 (f), or β4 (b, e). Data are means ± SEM of 5 (a–c) or 3 (e, f) independent experiments, each performed with duplicate or triplicate measurements. *Significantly different from zero (*p* < 0.05, one sample Student’s *t*-test). NS: not significantly different from zero (*p* > 0.05, one sample Student’s *t*-test).

We used sequential IP to assess the association of the subunits α3 and β4 in WT mice. In this set of experiments, our anti-α3 antibody precipitated 269.2 ± 28.3 fmol/mg [^3^H]-epibatidine-labelled receptors (*n* = 5, Fig. 2). When tissue extracts were first cleared with anti-β4 (the ‘clearing’ antibody, see Mao *et al.* 2008), the number of receptors precipitated from the residual supernatant with anti-α3 (as ‘capturing’ antibody) was significantly (*p* < 0.001, paired Student’s *t*-test) reduced to low but still measurable levels (15.9 ± 4.4 fmol/mg, *n* = 5, significantly different from zero, *p* < 0.05, one sample Student’s *t*-test, Fig. 2), suggesting that a small number of α3-containing receptors occur without β4. In fact, levels of α3 significantly exceeded β4 by 40.0 ± 9.7 fmol/mg (paired Student’s *t*-test, *p* < 0.01, *n* = 10) when α3- and β4-containing receptors were determined in one IP experiment, again suggesting that in the P18 WT mouse habenula, α3-containing receptors occur without β4.

In contrast, the anti-β4 antibody precipitated 234.3 ± 29.8 fmol/mg [^3^H]-epibatidine without pre-clearing, but only 5.8 ± 4.9 fmol/mg [^3^H]-epibatidine after clearing with anti-α3 (*n* = 5, value not significantly different from zero, *p* > 0.05, one sample Student’s *t*-test, Fig. 2). These observations suggest that the great majority of receptors are of the α3β4* type but that a small number of α3β2* receptors exist as well. α3β2 receptors in the medial habenula have previously been shown with [^125^I]α-conotoxin MII autoradiography (Whiteaker *et al.* 2002).

### A significant number of receptors in the mouse habenula contain both β2 and β4 subunits

A significant number of receptors in the P18 mouse habenula appears to contain both β2 and β4 subunits, because the algebraic sum of receptors precipitated by either anti-β2 or anti-β4 antibodies significantly exceeds 100% (by 18% or 56 fmol/mg, *n* = 11, paired Student’s *t*-test, *p* < 0.001). This finding is supported by the observation that after pre-clearing with anti-β4, β2 levels are reduced by 65.7 ± 2.9 fmol/mg (*n* = 3, Fig. 2f). In contrast, β4 levels are reduced by 108.2 ± 4.9 fmol/mg after pre-clearing with anti-β2 (*n* = 3, Fig. 2e).

### α5-containing receptors include the subunits β2 and β4

**Fig. 3:**
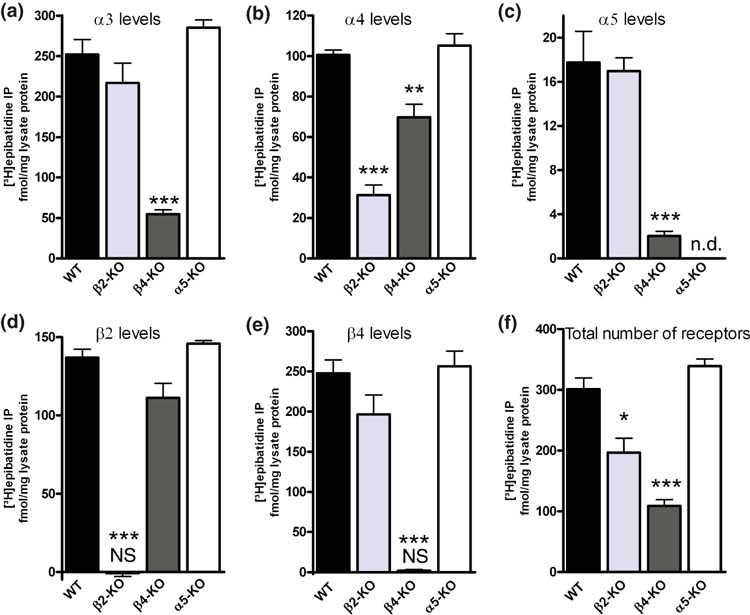
Levels of nAChR in habenulae of wild-type and indicated null mice. nAChRs from habenula of wild-type mice and of mice lacking distinct nAChR subunit genes (indicated at the abscissa) were solubilized, labeled with 1 nM [^3^H]-epibatidine and immunoprecipitated with the subunit-specific antibodies against α3 (a), α4 (b), α5 (c), β2 (d), or β4 (e). The total number of receptors in panel (f) was judged by a combined precipitation of anti-β2 and anti-β4 antibodies for WT and α5-KO mice, and by precipitation with anti-β4 and anti-β2 antibodies in β2-KO and β4-KO mice, respectively. Non-specific binding was measured in the presence of 300 μM nicotine and subtracted from the overall to obtain the specific binding shown in the figure. Data are means ± SEM of 3–6 (KO) or 6–22 (WT) independent experiments, each performed with triplicate measurements. Column data were compared using one-way anova followed by Bonferroni’s *post hoc* multiple comparison test. Significantly different from WT with *p < 0.05, **p < 0.005, ***p < 0.001. n.d.: not determined. NS: not significantly different from zero (*p* > 0.05, one sample Student’s *t*-test).

We next investigated the possible association of the α5 subunit with the subunits β2 and β4 by sequential IP. As shown in Fig. 2c, the anti-α5 antibody precipitated 21.5 ± 5.4 fmol/mg [^3^H]-epibatidine-labeled receptors. Upon pre-clearing extracts with anti-β4 in paired experiments, the receptors precipitated with anti-α5 dropped to levels not significantly different from zero (2.1 ± 1.3 fmol/mg, *n* = 5, *p* > 0.05, one sample Student’s *t*-test). Likewise, levels of α5-containing receptors fell from 18.7 ± 5.3 fmol/mg to 2.2 ± 3.4 fmol/mg after clearing with anti-β2, indicating an association of α5 and β2 as well (*n* = 5, not significantly different from zero, *p* > 0.05, one sample Student’s *t*-test, Fig 2d). As all β4 subunits co-assemble with α3 into one receptor (Fig. 2b), these observations suggest a receptor consisting of α3α5β4β2. It should, however, be noted that any protocol that causes a reduction of α5-containing receptors that are already expressed at low levels pushes measurements to the limits of detection. Therefore these conclusions must be treated with caution. Interestingly, α5-containing receptors are unaffected in β2 null mice but are almost eliminated in the β4 KO (Fig. 3c), showing that α5 requires the presence of β4 but not of β2 for assembly. As some α5-containing receptors remain in β4 null mice (2.0 ± 0.4 fmol/mg, *n* = 5, *p* < 0.01, one sample Student’s *t*-test, significantly different from zero; Fig. 3c) a few receptors may assemble from just α3, α5, and β2 in this genotype. Still, deletion of the α5 subunit did not affect the expression of nAChRs at large or receptors containing the subunits α3, α4, β2, or β4 (Fig. 3).

### Two major entities of nAChRs in the mouse habenula: α3β4*, and α4β2*: Evidence from KO models

Results from mouse models with deletions of the β2 or the β4 subunit nicely match our IP experiments with anti-β2 and anti-β4 antibodies. Whereas an IP using anti-β2 precipitates 46% of WT receptors (Fig. 1), 36% of overall receptors are also lost in β2 null mice (Fig. 3f). Likewise, an IP using anti-β4 precipitates 84% of receptors in WT (Fig. 1), while 67% of overall receptors are lost in the β4 KO (Fig. 3f). The great majority of the α3 subunit appears to co-assemble with β4, because levels of α3-containing receptors were largely reduced (by about 80%) – but not eliminated – in the β4 KO model (Fig. 3a).

About 70% of nAChRs containing the α4 subunit are lost in the β2 KO model, indicating the extent to which α4β2 receptors are present in the WT mouse habenula (Fig. 3b). However, about 30% of α4-containing receptors are also lost in the β4 KO, implying that some α4 normally co-assembles with β4 (Fig. 3b), as previously suggested by Grady *et al.* (2009). We propose that the latter receptors also contain the subunit α3, because sequential IP with anti-α3 as the clearing antibody removes all β4-containing receptors in the supernatant (Fig. 2). The combined presence of α3 and α4 in one receptor is in keeping with the observation that the algebraic sum of receptors containing the subunits α3 and α4 significantly exceeds 100% (by 20% or 56 fmol/mg lysate protein, *n* = 5, paired Students *t*-test, *p* < 0.05).

It is worth noting that levels of β2- and β4-containing receptors are not significantly affected by deletions of the β4 and β2 subunit genes, respectively (Fig. 3d and e), indicating that the expression of these subunits is tightly regulated as in the mouse SCG (David *et al.* 2010).

## Discussion

Soon after the identification of neuronal-type nAChRs it became clear that a fairly large variety of such receptors are expressed at a remarkable density in the habenula, a pair of small nuclei above the thalamus (Wada *et al.* 1989; Hill *et al.* 1993; Zoli *et al.* 1998). More recently, α5-containing receptors in the habenulo-interpeduncular system have attracted considerable interest due to their involvement in controlling nicotine intake (Fowler *et al.* 2011; Frahm *et al.* 2011), in mediating nicotine withdrawal symptoms (Salas *et al.* 2009), and in affecting anxiety-related behavior (Gangitano *et al.* 2009). The subunit composition of hetero-pentameric nAChRs in the habenula has lately been analysed in rats, in WT mice, and in mice lacking the nAChR subunits β2 and β3 (Grady *et al.* 2009). Accordingly, the authors identified receptors that contain the subunits α2, α3, α4, α5, α6, β2, β3, and β4. The accessory subunit α5 co-assembled into both β2- and β4-containing receptors, even though levels of α5-containing receptors were not diminished in β2 KO mice (Grady *et al.* 2009). Whether α5 as an accessory subunit co-assembles with β2 or β4 is of importance, because the pharmacological and biophysical properties of β2- and β4-containing receptors differ significantly (Luetje and Patrick 1991; Fenster *et al.* 1997). α5 furthermore affects the function of both α3β4 (Gerzanich *et al.* 1998; Fischer *et al.* 2005; Frahm *et al.* 2011) and β2-containing (Ramirez-Latorre *et al.* 1996; Gerzanich *et al.* 1998) receptors. By re-assessing nAChRs occurring in the rodent habenula we therefore paid particular attention to α5-containing receptors and to which extent this subunit co-assembles with β2 and β4.

Our results are in keeping with previous observations that the habenula of mouse and rat is a rich source of nAChRs of great diversity. We found the overall number of hetero-oligomeric receptors (assessed by a combined use of β2 plus β4 antibodies), and their subunit composition is similar in rat and mice. Consistent with results by Grady *et al.* (2009) we precipitated receptors containing the subunits α3, α4, α5, β2, and β4. We also determined similar overall numbers of receptors in WT mice (301 versus 273 fmol/mg lysate protein by Grady *et al.* 2009), and we agree on the presence of major populations of α3β4* and α4β2*, an intermediate population of α3α4β4, and a minor population of α3β2 receptors. We were, however, unable to detect in P18 animals the low levels of α2 (2%) or α6 (3%) reported by Grady *et al.* (2009) in adult mice. Our sequential IPs indicate that all β4 co-assembles with α3, making α3β4* (about 85%) the most abundant nAChRs in the mouse habenula. In contrast, the data by Grady *et al.* (2009) suggest that β2-containing receptors (66%) outnumber β4-containing receptors (34%) in the adult mouse habenula. As we mostly used pre-pubertal animals, this discrepancy might be due to the age of the animals. However, we found more β4- than β2-containing receptors in adult mice as well, even though the frequency of β2 relative to β4-containing receptors increased by the age of the animals.

Previous observations in the rat MHb by autoradiography with [^125^I]-epibatidine in the presence or absence of ligands that block binding to α4β2 or α3β2 receptors estimated that α3β4-like binding accounts for > 85% of receptors, even though the authors caution that this value is almost certainly too high (Perry *et al.* 2002). In separate binding experiments using homogenates, Perry *et al.* (2002) maintain that α3β4-like [^125^I]-epibatidine binding still represented approximately 65% of the total sites. In keeping with this observation, binding in the rat MHb of [^3^H]-cytisine is relatively weak (40 fmol/mg protein) compared with [^125^I]-epibatidine binding (186 fmol/mg protein, Perry and Kellar 1995). Structures of the neocortex typically have a near 1 : 1 ratio of [^3^H]-epibatidine versus [^3^H]-cytisine binding (Perry and Kellar 1995). Because of its high affinity for most nAChRs, radiolabeled epibatidine has proven particularly useful for receptor autoradiography (see Perry and Kellar 1995; Zoli *et al.* 1998; Sharples *et al.* 2000; Whiteaker *et al.* 2000, 2002; Baddick and Marks 2011). Cytisine, on the other hand, has a higher affinity for α4β2- compared with α3β4-receptors and has been used at low concentrations to mask α4β2-containing receptors in epibatidine receptor autoradiography (see Perry *et al.* 2002; Baddick and Marks 2011).

Based on quantitative autoradiography, Zoli *et al.* (1998) also reported enriched binding of [^3^H]-epibatidine contrasting with much lower signals by both [^3^H]-nicotine and [^3^H]-cytisine in the mouse MHb. As [^3^H]-epibatidine binding was not reduced in β2 KO animals the authors conclude that ‘the vast majority of binding in this area consists of receptors that do not contain the β2 subunit’ (Zoli *et al.* 1998). Still, binding of [^3^H]-cytisine was diminished by > 50% in the MHb (medial part) in β2 KO mice. Furthermore, binding of [^125^I]-epibatidine measured autoradiographically in the MHb was unaffected not only in β2 but also in β4 KO mice (Baddick and Marks 2011), suggesting that autoradiography with radiolabeled epibatidine (in the absence of additional ligands) may not detect even significant losses (see our Fig. 3f and supplemental Table 3 by Grady *et al.* 2009) of overall nAChRs in the MHb of KO animals. Conversely, autoradiography with radiolabeled epibatidine in the presence of cytisine or A-85380 (a protocol which predominantly unveils β4-containing receptors, Perry and Kellar 1995; Perry *et al.* 2002; Whiteaker *et al.* 2000, 2002; Baddick and Marks 2011), shows significantly reduced binding in animals devoid of α3 (by 95%, Whiteaker *et al.* 2002) or β4 (by more than 75%, Baddick and Marks 2011). Although the experiments with KO animals conducted by Baddick and Marks (2011) demonstrate that both β2 and β4 are prominently expressed in the mouse MHb, they do not provide quantitative data on the presence of β4-relative to β2-containing receptors.

Functional experiments demonstrate that both β2- and β4-containing receptors are expressed in the habenula of rats and/or mice. Hence, patch clamp recordings from slices of the mouse ventromedial habenula show amplitudes of currents induced by 10 μM nicotine, 1,1-dimethyl-4-phenylpiperazinium, or cytisine in MHb neurons that do not differ between WT and β2 KO animals (Zoli *et al.* 1998). The moderate potency of the β2-preferring antagonist dihydro-β-erythroidine (Mulle *et al.* 1991), the pronounced inhibition by the α3β4-selective antagonist α-conotoxin AuIB in inhibiting nicotine-induced currents, and the relative potency and efficacy of cytisine (Quick *et al.* 1999) furthermore indicate the presence of α3β4-type somatic membrane receptors in acutely dissociated habenular neurons of the rat.

However, the acetylcholine-induced ^86^Rb^+^ efflux from mouse habenular synaptosomes is almost eliminated in β2 KO animals, suggesting that all pre-synaptic receptors contain the subunit β2 (Grady *et al.* 2009). Yet, whether β2- or β4 receptors make up the majority of the receptors in the rodent habenula cannot be deduced from these functional experiments. Taken together, with the exception of Grady *et al.* (2009), none of the above mentioned reports are in conflict, and some are in keeping with our own observation that the majority of receptors in the MHb contain the subunits α3 and β4.

**Fig. 4:**
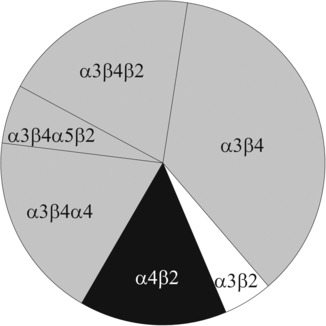
nAChRs occurring in the habenula of P18 wild-type mice. The diagram illustrates the proposed subunit composition of hetero-oligomeric receptors in P18 mice as deduced from data shown in Figs 1 and 2. The model is constructed from our observations that (a) the subunit β4 occurs only in combination with α3 (Fig. 2); (b) the subunit α5 occurs at an overall frequency of 6% (Fig. 1) and always co-assembles with α3β2β4 (Fig. 2); (c) some receptors contain both subunits α3 and α4, because the algebraic sum of receptors containing the subunits α3 and α4 significantly exceeds 100% (by 20% or 56 fmol/mg lysate protein, *n* = 5, paired Student’s *t*-test, *p* < 0.05); (d) the subunits β2 and β4 may also occur in one and the same receptor, because the algebraic sum of receptors containing the subunits β2 and β4 significantly exceeds 100% (by 18% or 56 fmol/mg, *n* = 11, paired Student’s *t*-test, *p* < 0.001), and because sequential IP with either anti-β2 or anti-β4 as clearing antibodies also removes significant quantities of β4- and β2-containing receptors, respectively (Fig. 2). The model is compatible with our two observations that (e) a small number of α3-containing receptors remains in the β4 KO (they assemble with β2, similar to the mouse SCG, see David *et al.* 2010); (f) about 30% of α4-containing receptors are lost in the β4 KO, and about 30% of α4-containing receptors remain in the β2 KO (Fig. 3), indicating that α4 co-assembles not only with β2 but also with β4.

Receptors that contain the accessory subunit α5 rely on the presence of β4 for assembly in the habenula, as shown by our observation that the great majority of α5-containing receptors are lost in β4 null animals. However, our sequential IPs suggest that β2 is also an integral part of α5-containing receptors. The existence of α3α5β4β2 receptors is in keeping with observations by Grady *et al.* (2009) who reported that in rats, immunodepletion with a β2-specific antibody significantly reduced the level of α5-containing receptors. We can also confirm the findings in this report that the number of α5-containing receptors is unaffected in β2 KO mice. Interestingly, ^86^Rb^+^ efflux from habenular synaptosomes induced by acetylcholine is largely reduced not only in β2 KO (Grady *et al.* 2009) but also in α5-KO mice (Fowler *et al.* 2011), suggesting receptors that contain both subunits. Still, the great majority of α3β4* receptors do not contain α5, and deletion of the α5 subunit gene does not affect the expression levels of either α3 or β4. Figure 4 illustrates nAChRs occurring in the habenula of mice as deduced from the data shown in Figs 1 and 2.

With 6% in P18 mice and 2.5% in P18 rats, α5-containing receptors comprise only a small fraction of overall receptors in the habenula. These levels are significantly lower than has previously been reported (8.5% in mice; 27.6% in rats, Grady *et al.* 2009) which suggests either a developmental regulation, that the efficacy of our anti-α5 antibody is too low, or that the IPs by Grady *et al.* (2009) detect an undue high number of α5-containing receptors. To address the first alternative we investigated the expression of α5-containing receptors in adult rats but found only a moderate increase (from 2.5% to 3.2% of overall receptors). In order to check the efficacy of our anti-α5 antibody we created a chimera where the cytoplasmic loop of the β2 subunit was replaced by the corresponding region of α5 (see Materials and methods). When expressed together with α4, the resulting receptors were precipitated equally well with both our anti-α5 and anti-α4 antibody, suggesting that the anti-α5 antibody has an efficiency > 90% (Figure S1). In native tissue, our antibody precipitated 24% and 20%α5-containing receptors in the mouse (David *et al.* 2010) and rat SCG (P. Scholze, unpublished observation), respectively. These data are in general agreement with observations by Mao *et al.* (2006) who reported 25–30% of α5-containing receptors in the rat SCG.

α5-containing receptors are important players in controlling addictive (Salas *et al.* 2009; Fowler *et al.* 2011; Frahm *et al.* 2011) as well as anxiety-related behavior (Gangitano *et al.* 2009). The altered anxiety-related response in mice lacking the β4 subunit (Salas *et al.* 2003) might therefore be due to missing α3β4* receptors which contain α5 as well. How these receptors mediate the behavioral effects is unclear at present. Single-cell RT-PCR suggests that the α3, α5, β2, and β4 subunits may be present in all cultured rat habenular neurons (Sheffield *et al.* 2000). These observations do not point at a particular cell type where somatic α3α5β4β2 receptors are concentrated to serve a specific function.

Alternatively, α5-containing receptors might preferentially be targeted to distinct axonal projections. Neurons in the MHb are cholinergic (Grady *et al.* 2009), glutamatergic (McGehee *et al.* 1995; Girod *et al.* 2000), or even both cholinergic and glutamatergic (Ren *et al.* 2011) and mainly project to the interpeduncular nucleus (Hikosaka 2010). Aversive high doses of nicotine activated the interpeduncular nucleus (IPN) in mice, reflected by increased Fos immunoreactivity (Fowler *et al.* 2011). This effect was almost completely abolished in α5 knockout mice, suggesting that α5-containing receptors support glutamatergic transmission in the IPN under these conditions (Fowler *et al.* 2011). However, nicotine applications facilitated the frequency of glutamatergic miniature excitatory post-synaptic potentials recorded from chick IPN neurons (co-cultured with explants of the habenula) if treated with α5-antisense-oligonucleotides (Girod *et al.* 2000). Recent experimental evidence furthermore suggests that the function of α3β4 receptors is reduced by the presence of α5 (and even more by its D397R variant, Frahm *et al.* 2011). These data are in line with our previous observations in mouse SCG neurons that deletion of α5 greatly enhances the outflow of [^3^H]-norepinephrine in response to pre-synaptic nAChR activation (Fischer *et al.* 2005). It is worth mentioning that ACh-induced release of [^3^H]-ACh from IPN synaptosomes is unaltered in α5 null mice, suggesting that α5-containing receptors may not reside on cholinergic axons that innervate the IPN (Grady *et al.* 2009).

To conclude, we have re-investigated the subunit compositions of hetero-oligomeric nAChRs in the habenula of mice and rats. We found that β4- clearly outnumbers β2-containing receptors and that although α5 requires the presence of β4 (but not β2) it assembles into a nAChR containing α3β4 as well as β2 in the mouse habenula. Our results explain previous observations that nicotine withdrawal symptoms are abolished both in α5 and β4 KO mice (Salas *et al.* 2004, 2009). Considering our observations it will also be interesting to see whether a loss of control in nicotine consumption occurs not only upon deletion of the α5 (Fowler *et al.* 2011) but also of the β4 subunit gene. Gene association studies have linked nicotine abuse to gene variants of the *CHRNA5*/*CHRNA3* gene cluster on chromosome 15 (Berrettini *et al.* 2008; Bierut *et al.* 2008). Hence, despite their rare occurrence, the α3α5β2β4 receptor may play a key role in controlling nicotine dependence.

## Abbreviations used

IP: immunoprecipitation
IPN: interpeduncular nucleus
KO: knockout
MHb: medial habenula
nAChR: nicotinic acetylcholine receptor
P18: postnatal day 18
SCG: superior cervical ganglion
WT: wild-type

## Supporting information

**Figure S1.** Confirmation of the subunitspecificity of anti-α2, anti-α5, and anti-α6 antibodies by recombinant and native nAChRs.

As a service to our authors and readers, this journal provides supporting information supplied by the authors. Such materials are peer-reviewed and may be re-organized for online delivery, but are not copy-edited or typeset. Technical support issues arising from supporting information (other than missing files) should be addressed to the authors.
