# Supplementary material for: Subunit composition of α5-containing nicotinic receptors in the rodent habenula
Source: J Neurochem. 2012 May;121(4):551–60. doi: 10.1111/j.1471-4159.2012.07714.x (PMC3350326; doi:10.1111/j.1471-4159.2012.07714.x)
Supplement: Supplementary file 1 [file jnc0121-0551-SD1.pdf]

Supplemental material

<u>Supplemental Fig. 1:</u> Confirmation of the subunit specificity of anti-α2, anti-α5, and anti-α6 antibodies by recombinant and native nAChRs

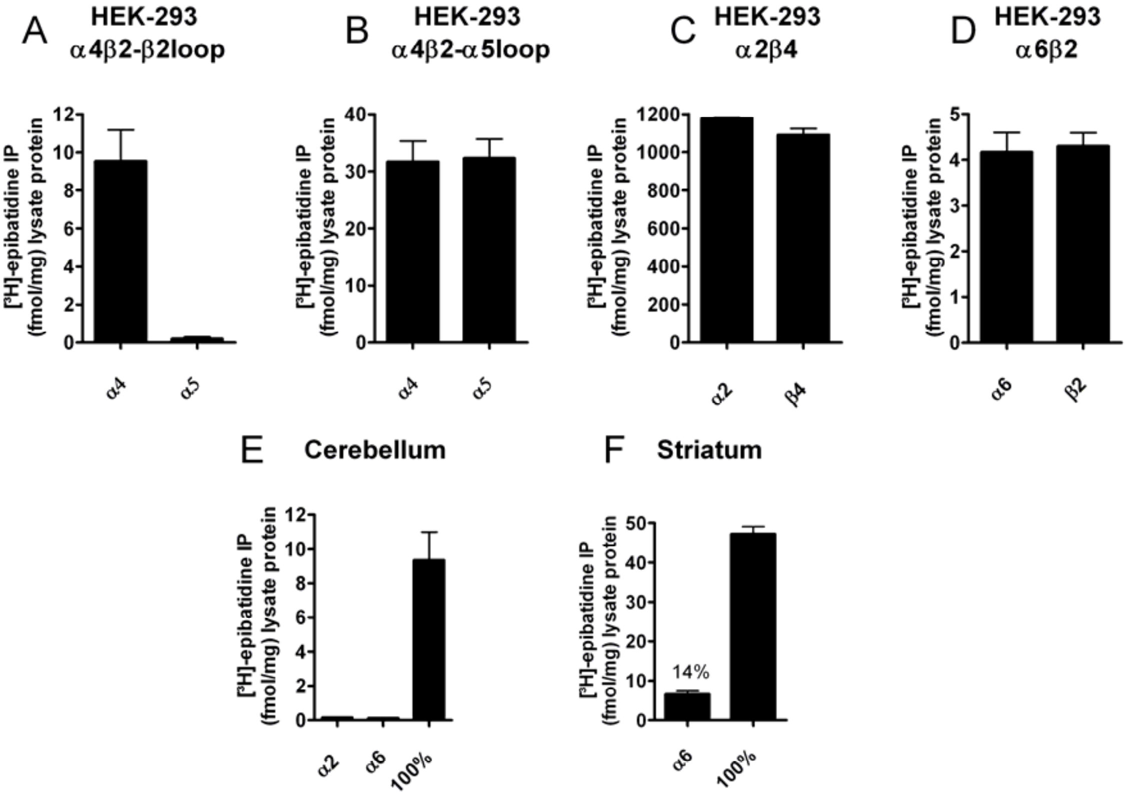

nAChRs from transfected HEK-293 cells, mouse cerebellum, or from striatum were solubilized, labeled with 1 nM [³H]-epibatidine, and immunoprecipitated with one of our antibodies (as indicated in the abscissa). The overall number of [^{3}H]-epibatidine binding sites in cerebellum or striatum ("100%") was determined by combined precipitations with anti-β2 plus anti-β4 antibodies (David *et al.* 2010).

(A) HEK-293 cells transfected with the subunits α4 and β2. Note that the anti-α5-antibody did not precipitate any receptors.

(B) HEK-293 cells transfected with α4 and a chimeric β2 subunit (the loop region of the β2 being replaced by the homologous α5 sequence, corresponding to the region used for antibody generation). The anti-α5 antibody now precipitates equal amounts of receptors as the anti-α4 antibody. The anti-α4 and anti-β2 antibodies have been characterized previously (David *et al.* 2010).

(C) HEK-293 cells transfected with the subunits α2 and β4. Note that the anti-α2-antibody

precipitates equal amounts of receptors as the anti-β4 antibody which has been characterized previously (David *et al.* 2010).

(D) HEK-293 cells transfected with the subunits α6 and β2. Note that the anti-α6 antibody precipitates equal amounts of receptors as the anti-β2 antibody.

(E, F) Assays of the anti-α2 and anti-α6 antibodies in native tissue. We used cerebellum (E) as a negative control (Panel E), since this tissue is known not to express the subunits α2 and/or α6 (Turner and Kellar 2005). Striatum was used as native positive control (Panel F), since it is one of the few areas in the brain rich in α6-containing receptors (Zoli *et al.* 2002; Champtiaux *et al.* 2003). Similar to published data (6.8 fmol/mg, Grady *et al.* 2009) our antibody detects between 15.4 and 36.1 fmol/mg α2-containing receptors in the interpeduncular nucleus (n = 2, unpublished observation).

Methods

Receptors were solubilized from transfected HEK-293 cells (Panels A, B, C, and D), cerebellum (Panel E), or striatum (Panel F) as described in Methods ("Immunoprecipitation of [^{3}H]-epibatidine labeled receptors").

Transfection protocol: Human embryonic kidney 293 (HEK-293) cells from American Type culture collection (Rockville, MD) were maintained in Dulbecco's Modified Eagle Medium (D-MEM, high glucose including GlutaMAX) supplemented with 100 units/ml penicillin and 100 µg/ml streptomycin, non-essential amino acids (all components from Invitrogen-Gibco) and 10% FCS at 5% CO_2 and 36.5° C. 1.8 x 10^6 cells were seeded onto a 10 cm tissue culture dish (Nunc) and transfected with 20 µg plasmid DNA via the calcium phosphate precipitation technique (Chen and Okayama 1987). Cells were harvested 44 hr after transfection. The generation of nAChR constructs (mouse subunit cDNA in pCI expression vector) has been described previously (Putz *et al.* 2008).

Reference List to the Supplemental Material

Champtiaux N., Gotti C., Cordero-Erausquin M., David D. J., Przybylski C., Lena C., Clementi F., Moretti M., Rossi F. M., Le Novere N., McIntosh J. M., Gardier A. M. and Changeux J.-P. (2003) Subunit composition of functional nicotinic receptors in dopaminergic neurons investigated with knock-out mice. *J. Neurosci.* **23,** 7820-7829.

Chen C. and Okayama H. (1987) High-efficiency transformation of mammalian cells by plasmid DNA. *Mol. Cell. Biol.* **7,** 2745-2752.

David R., Ciuraszkiewicz A., Simeone X., Orr-Urtreger A., Papke R. L., McIntosh J. M., Huck S. and Scholze P. (2010) Biochemical and functional properties of distinct nicotinic

acetylcholine receptors in the superior cervical ganglion of mice with targeted deletions of nAChR subunit genes. *Eur. J. Neurosci.* **31,** 978-993.

Grady S. R., Moretti M., Zoli M., Marks M. J., Zanardi A., Pucci L., Clementi F. and Gotti C. (2009) Rodent habenulo-interpeduncular pathway expresses a large variety of uncommon nAChR subtypes, but only the $\alpha3\beta4^*$ and $\alpha3\beta3\beta4^*$ subtypes mediate acetylcholine release. *J. Neurosci.* **29,** 2272-2282.

Putz G., Kristufek D., Orr-Urtreger A., Changeux J. P., Huck S. and Scholze P. (2008) Nicotinic acetylcholine receptor-subunit mRNAs in the mouse superior cervical ganglion are regulated by development but not by deletion of distinct subunit genes. *J. Neurosci. Res.* **86,** 972-981.

Turner J. R. and Kellar K. J. (2005) Nicotinic cholinergic receptors in the rat cerebellum: multiple heteromeric subtypes. *J. Neurosci.* **25,** 9258-9265.

Zoli M., Moretti M., Zanardi A., McIntosh J. M., Clementi F. and Gotti C. (2002) Identification of the nicotinic receptor subtypes expressed on dopaminergic terminals in the rat striatum. *J. Neurosci.* **22,** 8785-8789.
